# Does balloon tamponade really make postpartum haemorrhage worse?

**DOI:** 10.1111/1471-0528.15948

**Published:** 2019-10-03

**Authors:** AD Weeks

**Affiliations:** ^1^ Sanyu Research Unit Liverpool Women's Hospital and University of Liverpool for Liverpool Health Partners Liverpool UK

The Anger paper examines the benefits of introducing condom balloon tamponade (CBT) into hospitals in low‐income settings (Anger et al. *BJOG* 2019;126:1612–21). It follows on from the only other randomised controlled trial of CBT, which showed, much to everyone's surprise, a worsening of postpartum haemorrhage (PPH) outcomes with use of CBT in health centres (Dumont et al. *BMJ Open* 2017;7:e016590). Indeed, this much larger study was conducted primarily to disprove the results of the first. But instead, it has supported them: the rate of PPH‐related surgery and death nearly doubled with the introduction of the device. This is causing concern for those who are already implementing the CBT based on high‐quality cohort studies (Burke et al. *BJOG* 2016;123:1532–40). It also provides a dilemma for those of us who use and value commercial balloon tamponade devices in our own clinical practice.

The first question is whether balloon tamponade is effective at all at stopping bleeding. The Anger and Dumont studies both used a saline‐filled condom tied to a Foley catheter. This is low cost, but may produce inadequate intrauterine pressures (Antony et al. *AJP Rep* 2017;7:e86–e92). In this study, we cannot blame the device directly for the poor outcomes because 78% of the women with poor outcomes did not have CBT used at all. The efficacy question is therefore left hanging – is the problem the setting, the device or the technique itself? The first job is to test the technique, and a large randomised controlled trial of a commercial balloon device in a well‐functioning health system is being planned.

But why was the CBT not used more frequently? Analysis of maternal deaths shows that PPH deaths are rarely due to simple atony. Although an atonic uterus after uncomplicated vaginal birth usually responds well to uterotonics, PPH secondary to placental problems (abruption, accreta or praevia) or surgery is complex and requires well‐resourced operating facilities, skilled surgeons and blood (Weeks *BMJ* 2015;351:h3251). The use of CBT, even if it works well, is not and cannot be the only answer.

The authors suggest that the worsened outcomes were due to ‘temporal changes’. It is no surprise that CBT implementation more than doubled the rate of surgical interventions (from 11 to 26), probably due to the sensitisation and training that occurred at the study launch. However, surgery for collapsed, hypovolaemic women is extremely risky in under‐staffed theatres with limited access to blood. In this study, the procedure‐related mortality was 18% during the control period and 15% in the intervention period. Without regular supplies of blood and without improvements in theatre staffing and resources, the increased surgery may have killed more than it saved.

This study emphasises how deaths from PPH in low‐income settings are a multisystem healthcare problem. Any solution needs to address multiple problems simultaneously. Staffing, referral systems, infrastructure, consumables, devices, training and support, corruption, blood and supply chains all need to be improved if maternal mortality is going to decrease. Once again, we see that there are no magic bullets and, sadly, no short cuts.

## Disclosure of interests

Dr Weeks reports grants from PPH Butterfly, during the conduct of the study; in addition, Dr Weeks has a patent PPH Butterfly pending. A completed disclosure of interest form is available to view online as supporting information.
